# Estimating the cost-effectiveness of an infant 13-valent pneumococcal conjugate vaccine national immunization program in China

**DOI:** 10.1371/journal.pone.0201245

**Published:** 2018-07-25

**Authors:** Kunling Shen, Matthew Wasserman, Dongdong Liu, Yong-Hong Yang, Junfeng Yang, Greg F. Guzauskas, Bruce C. M. Wang, Betsy Hilton, Raymond Farkouh

**Affiliations:** 1 Department of Respiratory Diseases, Beijing Children's Hospital, Capital Medical University, National Center for Children's Health, Beijing, China; 2 Pfizer Inc. New York, NY, United States of America; 3 Pfizer Inc., Beijing, China; 4 Key Laboratory of Major Diseases in Children and National Key Discipline of Pediatrics (Capital Medical University), Ministry of Education, National Clinical Research Centre for Respiratory Diseases, Beijing Key Laboratory of Pediatric Respiratory Infection Diseases, Laboratory of Microbiology, Beijing Children's Hospital, Beijing Pediatric Research Institute, Capital Medical University, Beijing, China; 5 Elysia Group, LLC., New York, NY, United States of America; 6 Pfizer Inc. Collegeville, PA, United States of America; Universidad Nacional de la Plata, ARGENTINA

## Abstract

**Background:**

The burden of pneumococcal disease in China is high, and a 13-valent pneumococcal conjugate vaccine (PCV13) recently received regulatory approval and is available to Chinese infants. PCV13 protects against the most prevalent serotypes causing invasive pneumococcal disease (IPD) in China, but will not provide full societal benefits until made broadly available through a national immunization program (NIP).

**Objective:**

To estimate clinical and economic benefits of introducing PCV13 into a NIP in China using local cost estimates and accounting for variability in vaccine uptake and indirect (herd protection) effects.

**Methods:**

We developed a population model to estimate the effect of PCV13 introduction in China. Modeled health states included meningitis, bacteremia, pneumonia (PNE), acute otitis media, death and sequelae, and no disease. Direct healthcare costs and disease incidence data for IPD and PNE were derived from the China Health Insurance and Research Association database; all other parameters were derived from published literature. We estimated total disease cases and associated costs, quality-adjusted life years (QALYs), and deaths for three scenarios from a Chinese Payer Perspective: (1) direct effects only, (2) direct+indirect effects for IPD only, and (3) direct+indirect effects for IPD and inpatient PNE.

**Results:**

Scenario (1) resulted in 370.3 thousand QALYs gained and 12.8 thousand deaths avoided versus no vaccination. In scenarios (2) and (3), the PCV13 NIP gained 383.2 thousand and 3,580 thousand QALYs, and avoided 13.1 thousand and 147.5 thousand deaths versus no vaccination, respectively. In all three scenarios, the vaccination cost was offset by cost reductions from prevented disease yielding net costs of ¥29,362.32 million, ¥29,334.29 million, and ¥13,524.72 million, respectively. All resulting incremental cost-effectiveness ratios fell below a 2x China GDP cost-effectiveness threshold across a range of potential vaccine prices.

**Discussion:**

Initiation of a PCV13 NIP in China incurs large upfront costs but is good value for money, and is likely to prevent substantial cases of disease among children and non-vaccinated individuals.

## Introduction

*Streptococcus pneumoniae* is responsible for a significant burden of vaccine preventable disease, notably causing invasive pneumococcal disease (IPD) such as meningitis and bacteremia, and non-invasive forms of disease such as pneumonia (PNE) and acute otitis media (AOM). Furthermore, these diseases can result in numerous hospitalizations, outpatient visits, antibiotic prescriptions, and lead to productivity losses for parents and other caregivers [1]. The World Health Organization (WHO) estimates that IPD alone kills close to half a million children each year [2], with most of these deaths occurring in developing countries [3]. Prior to 2008, there were approximately 30,000 childhood (<5 year old) deaths each year in China due to pneumococcal disease, which accounted for approximately 12% of the total global pneumococcal disease burden [4].

Globally, pneumococcal vaccines (PCV) are proven to be a safe and effective intervention to prevent against invasive and non-invasive forms of the disease. The first PCV, a 7-valent pneumococcal conjugate vaccine (PCV7), offered protection against 7 pneumococcal serotypes (4, 6B, 9V, 14, 18C, 19F, 23F) and was first approved in the United States in 2000. In 2007, the WHO recommended incorporating the vaccine into national childhood immunization programs [2], and inclusion of PCV7 vaccines in national immunization programs (NIP) has led to a substantial decline in pneumococcal diseases in children and adults.

Since 2009, PCV7 has been replaced globally by next generation PCVs that provide additional serotype coverage. The 10-valent pneumococcal conjugate vaccine (PCV10) offers seroprotection against three additional serotypes (1, 5, and 7F) that are not covered in PCV7 and 13-valent pneumococcal conjugate vaccine (PCV 13) protects against six additional serotypes (1, 3, 5, 6A, 7F, and 19A) that are not covered in PCV7. Globally, the introductions of pneumococcal conjugate vaccines for infants and children have resulted in substantial declines in IPD, PNE, and AOM in countries that have implemented a NIP [5–9]. This has been coupled with a substantial herd effect, leading to reductions in disease both in vaccinated and unvaccinated children, as well as older age groups [10]. To date, over 100 countries have implemented national immunization programs with higher valent PCVs, leading to a beneficial clinical and economic impact both in vaccinated and unvaccinated populations.

Despite these benefits many countries in Asia, including China, have not adopted PCVs in their national immunization schedules due to the associated costs, logistical challenges of introducing a new vaccine, and a lack of disease burden data. PCV7 was approved in China in 2008, and while there was some uptake until the vaccine was discontinued in 2013, uptake was low and concentrated in cities [11, 12]. For this reason the burden of disease was expected to remain largely uninfluenced by vaccine. In 2016, the Chinese Food and Drugs Administration (CFDA) approved PCV13 for use in infants and children in a 3+1 schedule at 2, 4 and 6 months of age with a fourth (booster) dose administered at approximately 12–15 months of age [13]. However, like PCV7, PCV13 is currently only available to infants whose parents are able to self-pay for the vaccine. Because of limited uptake and recent introduction of PCV13, pneumococcal disease remains a significant burden, including disease caused by serotypes 19A (29%), 19F (22.5%) and 14 (12.9%)[14], all of which are covered by PCV13. Therefore, inclusion of PCV13 in the Chinese NIP is essential, given the significant burden related to pneumococcal diseases and the high incidence of disease caused by serotype 19A.

Numerous cost-effectiveness analyses of PCV13 have demonstrated that the introduction of a PCV13 NIP is cost-effective or cost-saving, particularly when the indirect effects are considered [15, 16], and several cost-effectiveness studies in China have shown that introducing PCV13 as part of the NIP is highly cost-effective [17, 18]. However, these analyses utilized epidemiologic and cost data from Taiwan as a proxy for the burden in China, leading to uncertainty as to the validity of study findings.

The goal of this study was to estimate the clinical and economic benefits of introducing the PCV13 vaccine into a NIP in China leveraging local epidemiologic and cost inputs, while also accounting for the robust indirect effects that PCV13 provides.

## Methods

### Approach

We adapted a well-established decision analytic model to predict the impact on the Chinese population following vaccination of a single birth cohort (19). This model has been adapted for use across a number of settings implementing a PCV13 program [19–23]. Infants enter the model with or without vaccine protection, dependent on assumed vaccine uptake, and then enter into one of the following mutually exclusive health states: no disease, meningitis, bacteremia, pneumonia (PNE), acute otitis media, and death. The model evaluates the number of cases averted, associated deaths and sequelae prevented, costs, life-years, and quality-adjusted life years. Outcomes were calculated over a 1-year, steady-state period considering direct and indirect protection afforded by vaccination. The steady state reflects a future period where the full benefits of a vaccine program are achieved. The Chinese government set up the goal of 85% uptake for all vaccines included in the NIP, and so far has achieved this goal [24, 25]. Therefore, in the NIP scenario, this assumed full NIP implementation in China with an 85% uptake by eligible infants across the entire population, and explored regional variation in scenario analyses. Vaccinated children received 4 doses of PCV13 in a 3+1 schedule as this is currently the only approved schedule in China. We calculated costs (in CNY) and outcomes based on a Chinese payer perspective, and discounted lifetime outcomes at 3% per annum.

### Epidemiologic inputs

Disease incidence data for IPD and PNE was derived from the China Health Insurance and Research Association (CHIRA) database and are found in Table 1 [26]. The CHIRA database gathers diverse information (e.g., patient characteristics, diagnosis, treatment, medication and costs) from health insurance offices/centers of selected areas around China, which covers 4 municipalities (Tier 1), all capital cities of each province (Tier 2), 1~2 prefecture-level cities (Tier 3) and 1~2 county level cities (Tier4) from each province. Sampling proportions were 2% in municipalities and capital cities of each province, 5% for prefecture-level cities, 10% for county-level cities. The sampling method for the CHIRA database is that patients are listed via descending ages and are subject to random equidistant sampling for records of medicine use and medical treatment over a full year in the following five steps:

1. Retrieval of each individual patient’s (based on Patient De-identified ID) from the local insurance offices/centers and eliminate repetitive information and obtain sampling pool.
2. Sorting of patients according to age in descending order and add sequence number to each patient (after sorting completed).
3. Calculation of sample interval. For example: A prefecture-level city, the sampling interval is 20 people with a sampling rate of 5% (1/5%).
4. Random identification of the first subject, and selection of all subjects by mechanical sampling.
5. Retrieval and collection of all information from the selected samples relevant for the analysis.

**Table 1:** Model parameters.

Model Parameter, By Age:	0 - < 2	2 - < 5	5–17	18–34	35–49	50–64	65+
Incidence/100,000*							
Pneumococcal Bacteremia	2.66 (2.00–3.33)	8.35 (5.60–10.44)	0.94 (0.71–1.18)	2.28 (0.30–2.85)	0.32 (0.24–0.40)	0.87 (0.30–1.09)	0.98 (0.74–1.23)
Pneumococcal Meningitis	1.25 (0.90–1.56)	0.58 (0.00–0.73)	1.10 (0.10–1.38)	0.64 (0.06–0.80)	0.24 (0.06–0.30)	0.20 (0.06–0.25)	0.10 (0.08–0.13)
Pneumonia-Inpatient	14,857 (3,929–25,784)	14,059 (1,743–26,375)	13,370 (10,028–16,713)	2,193 (1,645–2,741)	1,131 (848–1,414)	1,252 (939–1,565)	1,894 (1,421–2,368)
Pneumonia-Outpatient	2,165(1,624–2,706)	2,238 (1,679–2,798)	861 (645–1,076)	254 (190–317)	220 (165–275)	177 (133–221)	190 (142–237)
Otitis Media (Mild)	10,678(8,009–13,348)	10,678(8,009–13,348)					
Otitis Media (Moderate to Severe)	3,891 (2,918–4,864)	3,891(2,918–4,864)					
Case Fatality*							
Pneumococcal Bacteremia	4.0%	1.0%	5.0%	5.0%	6.0%	6.0%	6.0%
Pneumococcal Meningitis	14.0%	3.0%	4.0%	0.0%	12.0%	0.0%	2.0%
Pneumonia-Inpatient	0.5%	0.5%	0.5%	6.0%	6.0%	10.0%	16.0%
Direct Medical Cost*							
Pneumococcal Bacteremia	¥695	¥2,450	¥542	¥408	¥901	¥1,407	¥2,147
Pneumococcal Meningitis	¥25,602	¥5,315	¥6,967	¥13,105	¥5,988	¥9,345	¥6,463
Pneumonia-Inpatient	¥4,618	¥3,101	¥3,348	¥5,140	¥5,090	¥7,518	¥11,820
Pneumonia-Outpatient	¥195	¥205	¥194	¥253	¥277	¥353	¥430
Otitis Media	¥1,068	¥689					

*numbers in parentheses reflect values varied in sensitivity analysis as either: 95% CI, +/-10% or min/max from published literature in the case of inpatient pneumonia.

All case fatality rates and costs were varied based on +/-10% in sensitivity analysis.

While the incidence of hospitalized PNE extracted from the CHIRA database was within range of a recent systematic review in China [27], the rate derived from the CHIRA database was among the highest estimates found and may be the result of a classification error in the CHIRA database, i.e., inpatient-treated children who were treated multiple times during a period year may have been counted as different patients each time, or conditions coded as pneumonia could actually be a broader swath of respiratory ailments. To address this concern, we used estimates from a recent publication by Li, et al. (2017) [28] for the lower bounds for inpatient pneumonia ages 0–5, and used the CHIRA estimates as the upper bound; we then took the average between the two sources to derive the base case estimates. Data for IPD derived in the CHIRA dataset was consistent with incidence rates found in other studies [29]. Because limited AOM data were available within the CHIRA database, AOM incidence was taken from a previous PCV cost-effectiveness study conducted from the China perspective [30]. Sensitivity analysis ranges for incidence estimates were derived from either +/-10% of base case or the published literature. Sensitivity analysis was also conducted on uptake with a range of 70% to 95% based on national goal for vaccine coverage [31, 32]. A minimum of 70% was assumed given that a lower vaccine uptake may not be sufficient to generate indirect effects. Therefore, using an uptake rate below 70% may overestimate the benefits of a PCV program in the unvaccinated population and in such cases the direct effect only scenario should be referenced (scenario 1). Currently, no implicit threshold exists as a minimum number of infants needed to establish indirect protection for pneumococcal vaccination; however a study is currently ongoing to establish such a threshold in low and middle income countries and once this is available a concrete threshold could be used [33].

Age-specific life expectancy for healthy individuals was assumed to be the same as the general population. Case fatality data for individuals contracting IPD and inpatient PNE were obtained from previous PCV cost-effectiveness model estimates [17, 34]. Age-stratified bacteremia case fatality ranged from 1–6%, meningitis ranged from 0–14%, and inpatient PNE ranged from 1–16%. The only disease sequelae considered were in association with pneumococcal meningitis at an assumed rate of 7% for neurological impairment and 13% for hearing loss [35].

Serotype coverage estimates were derived from the published literature. For IPD, 87.1%, of disease was estimated to be caused by PCV13 serotypes based on a study of the serotype distribution and antimicrobial resistance patterns of *S*. *pneumoniae* isolates from children in China age <5 years [36]. For PNE, we used a similar study investigating the pneumococcal serotype distribution of children <5 years admitted to hospital with clinical pneumonia, which gave an estimate of 92.3% [37]. AOM serotype coverage (81.5%) was conservatively based on a multinational study of pneumococcal serotypes causing AOM in children [38]. The serotype distribution in adult IPD and pneumonia was assumed similar to that in children.

### Vaccine effectiveness

The model incorporates both the direct effect of reducing disease in vaccinated children, as well as indirect effects on adults and unvaccinated children as a result of herd effects (Table 2). The direct effect of vaccine coverage corresponds to the reduction in disease among vaccinated individuals. To estimate direct effects for PCV13, vaccine efficacy estimates from PCV7 clinical trial data were adjusted based on country-specific serotype coverage proportional to the additional serotypes covered in PCV13. Direct effects against IPD were assumed to be 94% versus covered serotypes for PCV13, based on clinical trial data for PCV7 [39] and immunogenicity trials showing comparable efficacy between PCV7 and PCV13 on shared serotypes. Direct effects against inpatient and outpatient pneumonia were derived from PCV7 all-cause disease estimates [35, 39] and were adjusted to country-specific and vaccine-specific serotype coverage. Estimates of efficacy against all-cause AOM were also similarly derived from PCV7 trial data [40].

**Table 2:** Direct and indirect effects for PCV13.

		0 - <2 years	2–4 years	5–17 years	18–34 years	35–49 years	50–64 years	65+ years
**Direct Effects (% reduction in disease at time of vaccination)**								
Pneumococcal Bacteremia	94%		94%	-	-	-	-	-
Pneumococcal Meningitis	94%		94%	-	-	-	-	-
Pneumonia-Inpatient	29%		29%	-	-	-	-	-
Pneumonia-Outpatient	7%		7%	-	-	-	-	-
Otitis Media (mild)	8%		8%	-	-	-	-	-
Otitis Media (moderate/severe)	17%		17%	-	-	-	-	-
Indirect Effects*								
Pneumococcal Bacteremia	64%		64%	53%	32%	32%	18%	12%
Pneumococcal Meningitis	64%		64%	53%	32%	32%	18%	12%
Pneumonia-Inpatient	22%		17%	0%	12%	5%	2%	3%

*Indirect effect adjusted in model to take into account vaccine coverage and interactions with direct effect to provide overall effect: Overall effect = [%vac x DE + 1-%vac*DE)*IDE] where %vac = percent vaccinated; DE = direct effect; and IDE = indirect effect.

Indirect effects represent the reduction in disease observed among unvaccinated individuals as a result of decreased carriage of *S*. *pneumoniae* among vaccinated individuals. Since the introduction of PCV13 seven years ago, numerous studies have demonstrated robust indirect effects. As PCV13 has been approved for the 3+1 schedule in China, data was used from the United States given its long history of PCV use and robust data available, and then adjusted by China-specific serotype coverage for PCV13 to estimate indirect effects in unvaccinated individuals [41–43]. The base case analysis assumed indirect effects for both IPD and inpatient PNE. Scenario analyses were undertaken to ascertain the robustness of the model under different indirect effect assumptions.

### Direct costs

All treatment-related cost data were derived from the CHIRA claims database. Direct medical costs were assumed to include hospitalization, physician consultations, diagnostic tests, surgeries, nursing, and medication expenses. The average price for the PCV13 vaccine was assumed to be 698 CNY per dose based on the current average list price, and we varied this by +/-15% in a price threshold analysis. While the analysis is focused on a full NIP, no public market price for PCV13 currently exists; in markets globally, the net price of vaccines procured for use in NIPs is substantially lower than list prices.

### Quality of life

Health-related quality of life is calculated by applying utility weights (scale: 0 = dead, 1 = perfect health) to survival. We assumed a general population utility for all healthy individuals of 0.9. Utility values were identified to estimate the quality adjusted life years (QALYs) for individuals experiencing a case of pneumococcal disease. QALY decrements were assumed for all disease states, representing the immediate short term impact of disease (bacteremia = 0.008; meningitis = 0.023; inpatient PNE = 0.006; outpatient PNE = 0.004; AOM = 0.005) [44], in addition to the utility decrement for meningitis sequelae of 0.40 for neurological impairment and 0.20 for hearing loss [45]. Acute disease QALYs were applied as a single decrement for each case of disease observed. As this is a 1-year steady state model, cumulative QALYs lost were calculated as cases times relevant QALY decrements. No individuals in this model are assumed to have repeat disease. Long term sequelae decrements were multiplied by the life expectancy of a patient at the time of the meningitis episode and discounted to estimate the current QALYs lost associated with long term sequelae.

### Analysis

For all outcomes generated from the model, life years, QALYs, and costs are calculated. Life years are estimated from the age distribution of the patient population and the conditional expected remaining life years by age group. Total QALYs are calculated as the product of discounted average life expectancy multiplied by the health utility index for a healthy patient. Incremental cost-effectiveness ratios (ICER) are then calculated as the difference between the expected costs between the two comparator cohorts divided by the difference in effectiveness between the two comparator cohorts. According to WHO guidelines, a medical intervention is cost-effective if its ICER is less than 3 times the per capita income. Given China’s gross national income (GNI) per capita of ¥53,976 in 2015, this threshold would be ¥161,929.

We calculated ICERs for the following three effectiveness scenarios: (1) direct effect only, (2) direct plus indirect effect for IPD only, and (3) direct plus indirect for IPDs plus inpatient PNE.

In addition to scenario analyses, one way sensitivity analyses were undertaken to test the robustness of results to uncertainty by varying all cost, incidence, CFRs, and utility parameters based on known confidence intervals, or a +/-10% range. A scenario was also evaluated varying vaccine uptake from 70% to 95%. This was only performed in the indirect effect scenarios as vaccine uptake does not impact ICERs when only considering direct effects because this is a steady state model and the impact proportionally falls relative to cost of the vaccine when excluding indirect effects.

## Results

The total number of pneumococcal disease cases and associated costs, QALYs, and deaths estimated in the model for all three scenarios are reported in Table 3. In the direct effect only scenario (1), implementing a PCV13 NIP in China gained 370.3 thousand QALYs and avoided 12.8 thousand deaths compared to no vaccination each year once a steady state has been achieved (Table 4). In the direct plus indirect effect for IPD only (2) and direct effect plus indirect effect for IPD and inpatient PNE (3) scenarios, the PCV13 NIP gained 383.2 thousand and 3,580.9 thousand QALYs, and avoided 13.1 thousand and 147.5 thousand deaths compared to no vaccination, respectively. In all three scenarios, the total vaccination cost for a PCV13 NIP in China was ¥38,382.2 million, however this was offset by medical cost-savings from prevented disease of (1) -¥9,019.9 million, (2) -¥9,047.9 million, and (3) -¥24,857.5 million, yielding total net costs of ¥29,362.3 million, ¥29,334.3 million, and ¥13,524.7 million, respectively. The resulting ICERs were (1) ¥79,304, (2) ¥76,551, and (3) ¥3,777, respectively. According to WHO guidelines, all three base case vaccine effectiveness scenarios above would be cost-effective and favorable relative to no vaccination compared to 3 times national GDP at the current list price.

**Table 3:** Total cases and costs with and without a PCV13 program.

Outcomes	No Vaccination	DE Only (1)	DE + IDE for IPD Only (2)	DE + IDE for IPD + PNE (3)
**Cases of Pneumococcal Disease (in thousands)**				
Pneumococcal Bacteremia	24.2	20.8	14.7	14.7
Pneumococcal Meningitis	7.7	7.3	5.0	5.0
Pneumonia-Inpatient	50,615.3	48,216.5	48,216.53	44,742.1
Pneumonia-Outpatient	5,577.2	5,489.2	5,489.2	5,489.2
Otitis Media (mild)	7,365.7	6,930.2	6,930.2	6,930.2
Otitis Media (moderate/severe)	2,683.9	2,343.8	2,343.8	2,343.8
**Total Cases (in thousands)**	**66,274.0**	**63,007.9**	**62,999.5**	**59,525.0**
**Total Deaths (in thousands)**	**1,412.0**	**1,399.20**	**1,398.8**	**1,264.5**
**Total QALYs Lost (in thousands)**	**32,687.4**	**32,317.2**	**32,304.2**	**29,106.5**
**Costs (in millions)**				
Vaccine Cost	¥0	¥38,382.2	¥38,382.2	¥38,382.2
Pneumococcal Bacteremia	¥20.6	¥13.0	¥9.1	¥9.1
Pneumococcal Meningitis	¥84.0	¥79.0	¥55.0	¥55.0
Pneumonia-Inpatient	¥212,752.6	¥204,387.1	¥204,387.1	¥188,577.5
Pneumonia-Outpatient	¥1,297.9	¥1,280.1	¥1,280.1	¥1,280.1
Otitis Media (mild)	¥5,759.9	¥5,419.3	¥5,419.3	¥5,419.3
Otitis Media (moderate/severe)	¥2,236.4	¥1,953.0	¥1,953.0	¥1,953.0
**Total Cost (in millions)**	**¥222,151.3**	**¥251,513.6**	**¥251,485.6**	**¥235,676.0**

Results Assume 85% uptake of PCV13 at a national level

DE = Direct effect; IDE = indirect effect; IPD = invasive pneumococcal disease; PNE = pneumonia; QALY = quality-adjusted life year

**Table 4:** Incremental cases and costs.

Outcomes	DE Only (1) vs No Vaccination	DE + IDE for IPD Only (2) vs No Vaccination	DE + IDE for IPD + PNE (3) vs. No Vaccination
**Cases of Pneumococcal Disease (in thousands)**			
Pneumococcal Bacteremia	-3.3	-9.4	-9.4
Pneumococcal Meningitis	-0.4	-2.7	-2.7
Pneumonia-Inpatient	-2,398.8	-2,398.8	-5,873.2
Pneumonia-Outpatient	-87.9	-87.9	-87.9
Otitis Media (mild)	-435.6	-435.6	-435.6
Otitis Media (moderate/severe)	-340.1	-340.1	-340.1
**Total Cases Averted (in thousands)**	**-3,266.1**	**-3,274.5**	**-6,749.0**
**Deaths Averted (in thousands)**	**-12.8**	**-13.1**	**-147.5**
**QALYs Gained (in thousands)**	**370.3**	**383.2**	**3,580.9**
**Costs (in millions)**			
Vaccine Cost	¥38,382.2	¥38,382.1	¥38,382.2
Pneumococcal Bacteremia	-¥7.6	-¥11.5	-¥11.5
Pneumococcal Meningitis	-¥4.9	-¥29.0	-¥29.0
Pneumonia-Inpatient	-¥8,365.5	-¥8,365.5	-¥24,175.1
Pneumonia-Outpatient	-¥17.9	-¥17.8	-¥17.8
Otitis Media (mild)	-¥340.6	-¥340.6	-¥340.6
Otitis Media (moderate/severe)	-¥283.4	-¥283.4	-¥283.4
**Total Cost (in millions)**	**¥29,362.3**	**¥29,334.3**	**¥13,524.7**
**ICER**	**¥79,304**	**¥76,551**	**¥3,777**

Results Assume 85% uptake of PCV13 at a national level

DE = Direct effect; IDE = indirect effect; ICER = incremental cost-effectiveness ratio; IPD = invasive pneumococcal disease; PNE = pneumonia; QALY = quality-adjusted life

**Fig 1:**
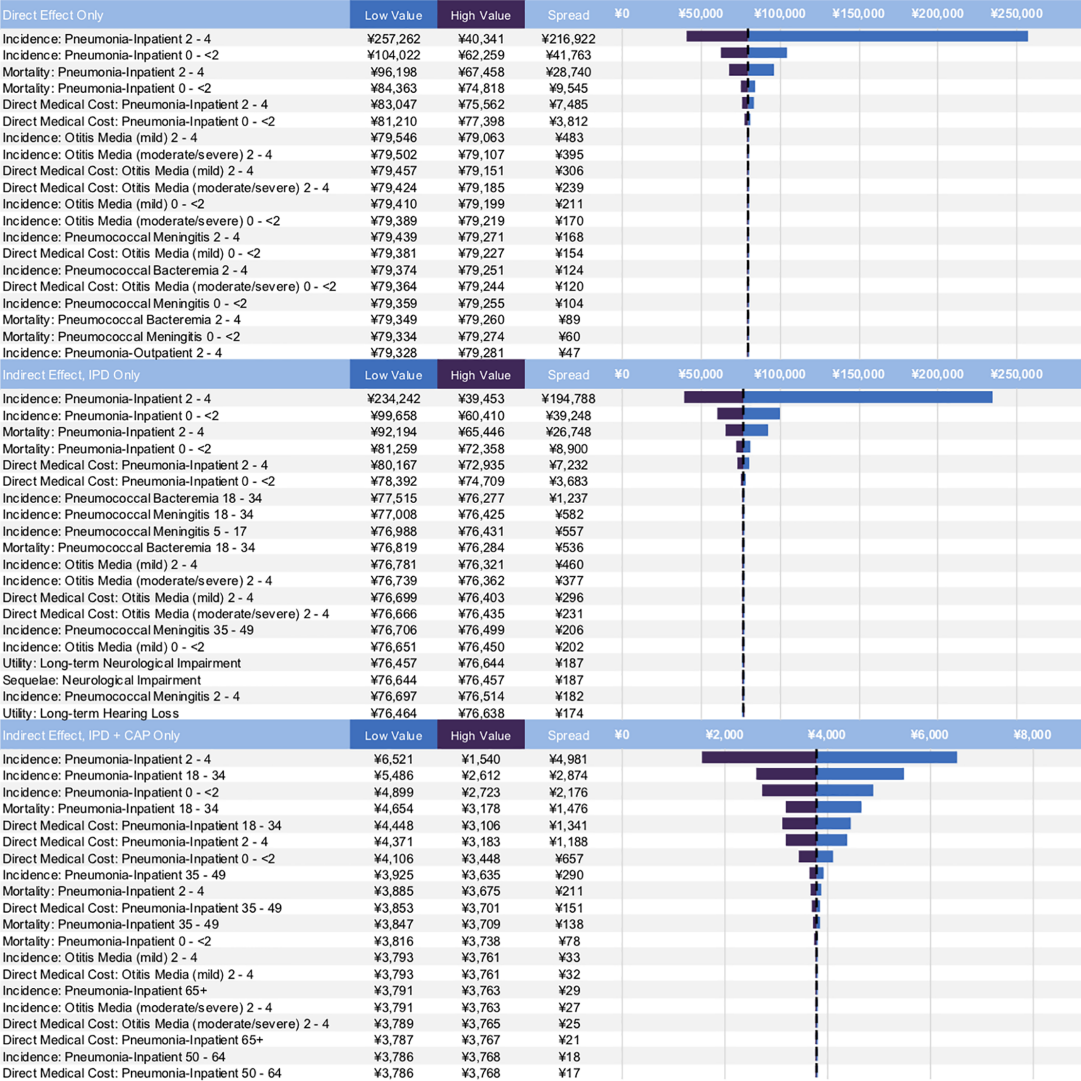
One way sensitivity analysis for most sensitive model parameters.

One-way sensitivity analyses for the top 20 most sensitive parameters are presented in Fig 1. Variation of the incidence rates to reflect the heterogeneity of China found that the incidence rates of inpatient PNE in ages 0–4 were the primary drivers of model uncertainty, with the low value of 2–4 year old incidence shifting the ICER to not cost-effective in scenarios 1 and 2. Ranging vaccine uptake from 70% to 95% were assumed to have no impact on scenario 1, and was found to have a limited impact in scenario 2 (¥75,906 to ¥76,871) and scenario 3 (¥2,286 to ¥4,746). Otherwise, a PCV13 NIP remained cost-effective when varying the remaining variables in the model.

As no public price exists for PCV13 in China, we assumed the current average list price of 698 CNY per dose and varied this cost by +/-15%. PCV13 was cost-effective at less than 2x GDP per capita across all price sensitivity threshold points for all three effectiveness scenarios: (1) ¥72,175 to ¥101,879, (2) ¥69,663 to ¥98,362, and (3) ¥3,040 to ¥6,111. When assuming direct effect plus indirect effect for IPD and inpatient PNE (scenario 3), PCV13 was highly cost-effective at less than 1x GDP across all price points. In scenario 3, PCV13 could be priced up to ¥3,917 per dose and remain cost-effective to the threshold of 1X GDP. Because China is a large and economically diverse country, we examined the ICERs relative to 32 provincial level GDPs and the cost-effectiveness thresholds these would imply. 31 out of 32 provinces remained cost-effective at 3X GDP in the most conservative scenario (1), and 4 provinces were considered highly cost-effective at 1X GDP. (S1 Table). In Scenario 3, PCV13 was cost-effective at 1XGDP in all provinces at 698 CNY per dose.

## Conclusion

We adapted a well-established PCV13 cost-effectiveness model to represent the implementation of a NIP in China. When considering direct vaccine effects only or indirect effects for rare invasive disease cases only, results were cost-effective at a threshold of 1–3 GDP per capita and remained cost-effective across a range of vaccine costs. When we included indirect effects for the more frequently occurring inpatient PNE, results are highly cost-effective at 1 times GDP per capita.

Our results indicate that a NIP with PCV13 would have a remarkable public health impact in China. Use of PCV13 would result in reductions in cases of disease and death, and due to cost-offsets following reductions in disease, would be highly cost-effective compared with no vaccination. Assuming our model adequately represents the Chinese population and health system; efforts should be made to ensure optimal implementation of a NIP in China for pneumococcal disease.

These results are consistent with economic evaluations recently published exploring the cost-effectiveness of PCV13 in China [17, 18]. However, this study adds to the body of evidence by utilizing local China data as opposed to data from Taiwan. For this reason, this study is likely more representative of the true cost-effectiveness of PCV13 in China. However, the CHIRA dataset primarily uses data from urban China, and rural China is not represented. It is believed that the burden of disease is higher in rural than in urban populations[27], but that the disease cost may be lower. For this reason, the ICERs generated in this study are likely still nationally representative.

The biggest challenge to this NIP-level analysis is the wide economic and epidemiologic variation across different provinces in China. Provinces may have considerably different serotype distributions, burden of pneumococcal disease, and costs related to treating disease. However a paucity of province specific data inhibits this understanding. Furthermore, given the disparity in income in China, the cost-effectiveness of the vaccine may warrant price variation at a provincial level to reflect local GDP per capita and be scaled up to a full NIP by first implementing Provincial Immunization Programs (PIPs). Assuming constant incidence of disease, at the current list price, and only including direct effects of the vaccine assuming potential low uptake in a PIP, PCV13 was estimated to be cost-effective at 3xGDP per capita across 31 out of 32 provinces, but only cost-effective at 1xGDP per capita in Beijing, Jiangsu, Shanghai, and Tianjin (See S1 Table). However, when including indirect effects for pneumonia, PCV13 is cost-effective at 1xGDP in all provinces. As mentioned in previous cost-effectiveness studies, despite being cost-effective, the price of the vaccine will have a significant budget impact in China, even at a provincial level. Given this heterogeneity of demographic, socio-economic, and epidemiologic factors and the costs of treatment in China evaluations should be undertaken at a provincial level to determine the precise cost-effectiveness of the vaccine. Despite this, even at the current average list price, we estimate that PCV13 is likely to be a highly cost-effective vaccine in PIPs across all of China.

Our model has a number of limitations worth noting. First, inpatient PNE data may greatly overestimate the true burden of disease, and was shown in sensitivity analysis to have a large impact on all model results. More research is needed to accurately account for the number of unique patients with PNE in China each year. Second, China is a large and diverse country, and our model attempts to generalize that diversity into a single cost-effectiveness ratio. Finally, the steady state model structure over a one year time horizon does not take into consideration potential serotype replacement in the long term and has limitations in the consideration of herd effects. However, the estimates of indirect PCV13 effects were based on ecologic studies that implicitly included emerging disease from non-vaccine serotypes so any bias should be minimized.

In conclusion, initiation of a PCV13 NIP in China incurs a high cost but is likely to have a substantial public health benefit. We estimated that a NIP with PCV13 has good value for money and prevents substantial disease among children and non-vaccinated individuals when compared to no pneumococcal vaccination. Our analyses may help inform policy makers of the potential benefits of vaccinating children in China, thereby reducing cases of preventable disease.

## Supporting information

S1 Table Cost-effectiveness of PCV13 across Chinese provinces at 698 RMB per dose, assuming only direct effects. (DOCX)
