# Supplementary material for: Estimating the cost-effectiveness of an infant 13-valent pneumococcal conjugate vaccine national immunization program in China
Source: PLoS One. 2018 Jul 25;13(7):e0201245. doi: 10.1371/journal.pone.0201245 (PMC6059448; doi:10.1371/journal.pone.0201245)
Supplement: S1 Table — (DOCX) [file pone.0201245.s001.docx]

**S1 Table: Cost-effectiveness of PCV13 across Chinese Provinces at 698 RMB per dose, assuming only direct effects**

| **Administrative Division** | **GDP per Capita** | **2x GDP per Capita** | **3x GDP per Capita** | **Cost-effective at 1x GDP per Capita** | **Cost-effective at 2x GDP per Capita** | **Cost-effective at 3x GDP per Capita** |
| --- | --- | --- | --- | --- | --- | --- |
| Tianjin | ¥ 107,960.00 | ¥ 215,920.00 | ¥ 323,880.00 | yes | yes | yes |
| Beijing | ¥ 106,497.00 | ¥212,994.00 | ¥319,491.00 | yes | yes | yes |
| Shanghai | ¥103,796.00 | ¥207,592.00 | ¥311,388.00 | yes | yes | yes |
| Jiangsu | ¥87,995.00 | ¥175,990.00 | ¥263,985.00 | yes | yes | yes |
| Zhejiang | ¥ 77,644.00 | ¥155,288.00 | ¥232,932.00 | No | yes | yes |
| Inner Mongolia | ¥71,101.00 | ¥142,202.00 | ¥213,303.00 | No | yes | yes |
| Fujian | ¥67,966.00 | ¥135,932.00 | ¥203,898.00 | No | yes | yes |
| Guangdong | ¥ 67,503.00 | ¥135,006.00 | ¥202,509.00 | No | yes | yes |
| Liaoning | ¥65,354.00 | ¥130,708.00 | ¥196,062.00 | No | yes | yes |
| Shandong | ¥64,168.00 | ¥ 128,336.00 | ¥192,504.00 | No | yes | yes |
| Chongqing | ¥52,321.00 | ¥104,642.00 | ¥156,963.00 | No | yes | yes |
| Jilin | ¥51,852.00 | ¥ 103,704.00 | ¥ 155,556.00 | No | yes | yes |
| Hubei | ¥50,654.00 | ¥101,308.00 | ¥151,962.00 | No | yes | yes |
| China | ¥50,251.00 | ¥100,502.00 | ¥150,753.00 | No | yes | yes |
| Shaanxi | ¥48,023.00 | ¥96,046.00 | ¥ 144,069.00 | No | yes | yes |
| Ningxia | ¥43,805.00 | ¥87,610.00 | ¥131,415.00 | No | yes | yes |
| Hunan | ¥ 42,968.00 | ¥85,936.00 | ¥ 128,904.00 | No | yes | yes |
| Qinghai | ¥41,252.00 | ¥ 82,504.00 | ¥ 123,756.00 | No | yes | yes |
| Hainan | ¥40,818.00 | ¥ 81,636.00 | ¥122,454.00 | No | yes | yes |
| Hebei | ¥40,255.00 | ¥80,510.00 | ¥120,765.00 | No | yes | yes |
| Xinjiang | ¥40,034.00 | ¥80,068.00 | ¥120,102.00 | No | yes | yes |
| Heilongjiang | ¥39,352.00 | ¥ 78,704.00 | ¥ 118,056.00 | No | No | yes |
| Henan | ¥ 39,131.00 | ¥78,262.00 | ¥117,393.00 | No | No | yes |
| Sichuan | ¥36,836.00 | ¥ 73,672.00 | ¥110,508.00 | No | No | yes |
| Jiangxi | ¥ 36,724.00 | ¥ 73,448.00 | ¥110,172.00 | No | No | yes |
| Anhui | ¥35,997.00 | ¥71,994.00 | ¥ 107,991.00 | No | No | yes |
| Guangxi | ¥35,190.00 | ¥70,380.00 | ¥105,570.00 | No | No | yes |
| Shanxi | ¥34,919.00 | ¥ 69,838.00 | ¥ 104,757.00 | No | No | yes |
| Tibet | ¥31,999.00 | ¥63,998.00 | ¥ 95,997.00 | No | No | yes |
| Guizhou | ¥29,847.00 | ¥ 59,694.00 | ¥ 89,541.00 | No | No | yes |
| Yunnan | ¥ 28,806.00 | ¥57,612.00 | ¥86,418.00 | No | No | yes |
| Gansu | ¥26,165.00 | ¥ 52,330.00 | ¥78,495.00 | No | No | No |

Ref: World Economic Outlook Database April 2016. (https://www.imf.org/external/pubs/ft/weo/2014/02/weodata/index.aspx)
